# Nasopharyngeal Carriage of *Streptococcus pneumoniae* Serotypes Among Healthy Children in Northern India

**DOI:** 10.1007/s00284-022-03114-x

**Published:** 2022-12-19

**Authors:** P. Gupta, S. Awasthi, U. Gupta, N. Verma, T. Rastogi, AK. Pandey, H. Naziat, H. Rahman, M. Islam, S. Saha

**Affiliations:** 1 Department of Microbiology, King George’s Medical University, Uttar Pradesh, Lucknow, India; 2 Department of Paediatrics, King George’s Medical University, Uttar Pradesh, Lucknow, India; 3 Department of Microbiology, Bangladesh Shishu Hospital & Institute, Dhaka, Bangladesh; 4 Child Health Research Foundation, Dhaka, Bangladesh

## Abstract

*Streptococcus pneumoniae* (SP) infections cause morbidity and mortality among children worldwide. Hence India introduced 13-valent pneumococcal conjugate vaccine (PCV-13) in 2017 in a phased manner. The primary objective of this study was to assess the proportion of healthy children having nasopharyngeal colonization (NP) with SP. Secondary objective was to determine prevalent serotype of SP among the PCV13 vaccinated and non-vaccinated children. This cross-sectional study was conducted in 4 hospitals of Lucknow District, Northern India. Three hundred healthy children (2–59 months) were recruited between July and August 2019 from vaccination-clinics of hospitals. NP specimen was cultured using 5% sheep blood agar plate containing gentamicin. Pneumococcal isolates were identified by optochin sensitivity and bile-solubility tests. Serotyping was done using Quellung Method. Of the 300 healthy children, 56.7% (170/300) were males and 59.3% (181/300) had received at least one dose of PCV13 vaccine. The NP carriage rate of SP among healthy children was 37.7% (113/300). Vaccine serotypes were found in 33.3% (22/66) in PCV vaccinated children and 48.9% (23/47) in non-vaccinated children (p 0.09). Common vaccine serotypes that isolated were: 18C, 19A, 19F, 23F, 3, 4, 6A, 6B, 9 V. Thus more than one-third of healthy children had NP colonization with SP. Adjusting for age, there was a trend for significant reduction in vaccine serotypes in the NP with one doses versus two or more doses (p_trend_ = 0.04).

**Supplementary Information:**

The online version contains supplementary material available at 10.1007/s00284-022-03114-x.

## Introduction

*Streptococcus pneumonaie* (SP) is one of the major bacteria responsible for causing various diseases such as otitis media, community acquired pneumonia, bacteraemia, meningitis, and sepsis [1, 2]_._ Worldwide, pneumococcal infection is a significant contributor to the under-five mortality [3]. Severe pneumococcal disease is most common in children under the age of 2 years. In 2018, globally 0.8 million children under age of 5 year died due to SP [4]. Most of these deaths occurred in low- and middle-income countries. In India, approximately 126,535 pneumococcal deaths occurred among under-five children in 2018 [4].

*Streptococcus pneumonaie* colonization in the nasopharynx plays an important role in the development of pneumococcal pneumonia and invasive pneumococcal disease (IPD). Most colonizations with SP in the nasopharynx of are asymptomatic [5]. About one-third of children and nearly 3–4% of adults are asymptomatically carriers of SP [6]. Many prior studies in India observed high nasopharyngeal (NP) colonization with SP [7–10]. The NP carriage of NP in children is affected by the environment as well as socio-economic factors like number of siblings, income, exposure to antibiotics, parental smoking, and day care center attendance [11–16]**.**

*Streptococcus pneumonaie*, a gram-positive bacterium, has 90 different serotypes [17, 18]**.** Pneumococcal vaccines have been developed against the most predominant serotypes causing IPD [19]. Pneumococcal vaccines, 10-valent (PCV10) and 13-valent (PCV13) are currently available in India. In the universal immunization program of the Government of India, PCV13 is being used and given at 6, 14, and 36 weeks of age since May 2017, PCV13 consists of serotypes 1, 3, 4, 5, 6A, 6B, 7F, 9 V, 14, 18C, 19A, 19F, and 23F [2]. Studies have reported that PCV13 noticeably decreased the incidence of pneumococcal diseases in children, due of serotypes 19A, 3, and 19F which are responsible for half of the cases [10, 20–23].

Since SP serotype distribution is crucial to evaluate the impact of new vaccine programs and help guide future vaccine formulations, our primary objective was to assess the proportion of healthy children having NP colonization with SP and the secondary objective was to determine prevalent serotype of SP among the PCV13 vaccinated and non-vaccinated groups.

## Study Methodology

This cross-sectional study was conducted from July to August 2019 in four tertiary care hospitals of Lucknow, Uttar Pradesh, North India. The NP swabs were collected from 300 healthy children, aged between 2 and 59 months. All the consecutive eligible children were recruited from the immunization clinic of the participating hospitals. Questionnaire was designed to collect information on the status of immunization and socio-demographic characteristics like gender, parents educational status and family type and anthropometric measurement of children. Family type defined as “nuclear, if the family had a nuclear pair comprising of head and spouse with or without unmarried children. A family that was not nuclear or single parent was considered joint” [24]. PCV13 vaccination data was abstracted from the immunization card of the children.

### Inclusion Criteria

Healthy children aged between 2 and 59 months who visited the immunization clinic of selected hospitals were included after obtaining written informed consent from their parents or legal guardians.

### Exclusion Criteria

We excluded those who were currently unwell, had been hospitalized in the last 3 months, and had received medications for any illness in the last 15 days or had been previously included in the survey.

### Sample Collection

Three hundred NP specimens were collected from children by sterile nylon flocked flexible swabs (HiMedia, India). Immediately swabs were placed in 1.0 ml skimmed milk-tryptone-glucose-glycerol transport (STGG) medium and placed in an ice box as per the World Health Organization’s consensus methods [25, 26]**.** Specimens were immediately transported to the microbiology laboratory for culture. All the samples were processed in the laboratory within 1 h.

### Laboratory Procedure

NP swabs were cultured on 5% sheep blood agar (Biomerieux, France) and 5% sheep blood agar with gentamycin (Himedia, India) for growth of SP and incubated in a candle jar at 37 °C for 18–24 h. All pneumococcal isolates were identified by standard microbiological methods [27–29]. All isolates were confirmed by optochin sensitivity and bile solubility tests. Isolates were identified as SP by colony morphology (Mucoid, draughtsman appearance, α-hemolysis) and susceptibility to optochin (positive was ≥ 14 mm diameter zone; negative was or < 14 mm of zone of inhibition), Those with optochin clearance zones was below 14 mm were further subjected to solubility in bile salts (positive as bile soluble; negative as bile insoluble).

In this study Matrix-assisted laser desorption/ionization time-of-flight (MALDI-TOF) Vitek Mass Spectrometry (Biomerieux) technique used for the correct identification of SP. Single colony was pick-up with sterile loop from the fresh bacterial culture and as a thin film smear made directly on the Maldi target plate. One microliter of a-Cyano-4-hydroxycinnamic acid (CHCA) *matrix* solution was then dropped into the smear. According to the manufacturer`s instruction, a confidence value from 60 to 99.9% of a species was taken for identification of SP. [30, 31].

### Serotyping of *S. pneumonaie*

Quellung reaction test was done by using SP isolates of fresh culture. A sterile loopful of the cells of fresh culture were suspended in 50ul of the 0.85% Saline to prepare a suspension. Subsequently, 2 ul of the suspended cells were added on to a glass slide and mixed with 5ul of pooled antiserum and 5ul methylene blue. These were mixed with a pipette tip. The suspension was covered with the cover slip and incubate at room temperature for 10–15 min. The glass slide was swirled gently while observing for any agglutination reaction until a positive reaction was observed with various pooled antisera. The process was repeated with individual groups with various antisera pools until a positive reaction with the particular serotype specific antisera was observed [32].

### Sample Size

Assuming that 20% [7] of children had NP carriage of SP, to calculate the minimum sample size, the following formula used was [7]:

$${\text{n}}\,{ = }\,\frac{{z^{2} \hat{p}\left( {1 - \hat{p}} \right)}}{{d^{2} }}$$

where, n = sample size, z = statistics corresponding to 95% level of confidence, $\widehat{p}$ = expected prevalence (20%), and d = precision (5%). The sample size required was 245. On the basis of this formula, we recruited 300 eligible children for this study.

### Data Management and Analysis

Clinical and laboratory data were entered online in customized Google doc file. Any child who had received even single dose of PCV13 was categorized as vaccinated. Univariate distribution of variables is being reported as frequency and percentage in tables as well as bar graph. Chi-square test without Yate’s correction was used for comparison of categorical variables, using statistical software SPSS (version 24, Chicago, Illinois, USA)**.** A p value of < 0.05 was taken as statistically significant using a two-tailed distribution. Logistic regression model for association of number of PCV doses of SP serotypes (vaccine vs non-vaccine) controlling for age. Chi- square for trend was used to see the linear association of PCV doses with the vaccine serotype.

## Results

This cross-sectional study was conducted in the month of July and August 2019. A total 300 healthy children, aged between 2 and 59 months were recruited. More than half (56.7%, 170/300) of the recruited children were male and their mean age was 16 ± 14 months. Table 1 shows the demographic characteristic of the subjects. More than three-fourths of those between 2 and 11 months of age had received at least one dose of PCV13. Almost all those who had received PCV13 were completely immunized for age.

**Table 1:** Socio-demographic Characteristics of the 300 healthy children tested for the nasopharyngeal carriage of *Streptococcus pneumoniae* by PCV vaccination status

Demographic characteristics of variables	Pneumococcal conjugate vaccination status			
	Overall *N* = 300(%)	With PCV *N* = 181(%)	Without PCV *N* = 119(%)	p value
Age in months				
2–11	156 (52.0)	139 (76.8)	17 (14.3)	< 0.001
12–23	92 (30.7)	29 (16.0)	63 (52.9)	
24–59	52 (17.3)	13 (7.2)	39 (32.8)	
Gender				
Male	170 (56.7)	103 (56.9)	67 (56.3)	0.92
Female	130 (43.3)	78 (43.1)	52 (43.7)	
Family type				
Joint	216 (72.0)	137 (75.7)	79 (66.4)	0.08
Nuclear	84 (28.0)	44 (24.3)	40 (33.6)	
Immunization status (excluding Pneumococcal Conjugate Vaccination)				
Complete for age	282 (94.0)	178 (98.3)	104 (87.4)	< 0.001
Incomplete/unimmunized	18 (6.0)	3 (1.7)	15 (12.6)	
Mother’s education				
No formal education/uneducated	20 (6.7)	7 (3.9)	13 (10.9)	0.02
Class I-X	66 (22.0)	37 (20.4)	29 (24.4)	
Class XI-XII	46 (15.3)	26 (14.4)	20 (16.8)	
Graduate	94 (31.3)	56 (30.9)	38 (31.9)	
Postgraduate	74 (24.7)	55 (30.4)	19 (16.0)	
Father’s education				
No formal education/uneducated	12 (4.0)	5 (2.8)	7 (5.9)	0.004
Class I-X	78 (26.0)	34 (18.8)	44 (37.0)	
Class XI-XII	48 (16.0)	33 (18.2)	15 (12.6)	
Graduate	98 (32.7)	66 (36.5)	32 (26.9)	
Postgraduate	64 (21.3)	43 (23.8)	21 (17.6)	
Nasopharyngeal Carriage of *Streptococcus pneumonaie*				
Yes	113 (37.7)	66 (36.5)	47 (39.5)	0.596
No	187 (62.3)	115 (63.5)	72 (60.5)	

### Carriage of *SP* Serotypes

Of the 300 healthy children, 37.7% (113/300) had NP carriage of SP. Of these, 36.5% (*n* = 66) had received PCV13 while 39.49% (*n* = 47) had not. There was no significant difference in NP carriage of SP between the vaccinated and non-vaccinated group (*p =* 0.596) (Table 1). The rate of colonization of SP was higher in male than in female children (58.4% vs 41.6%).

**Fig. 1 and 2:**
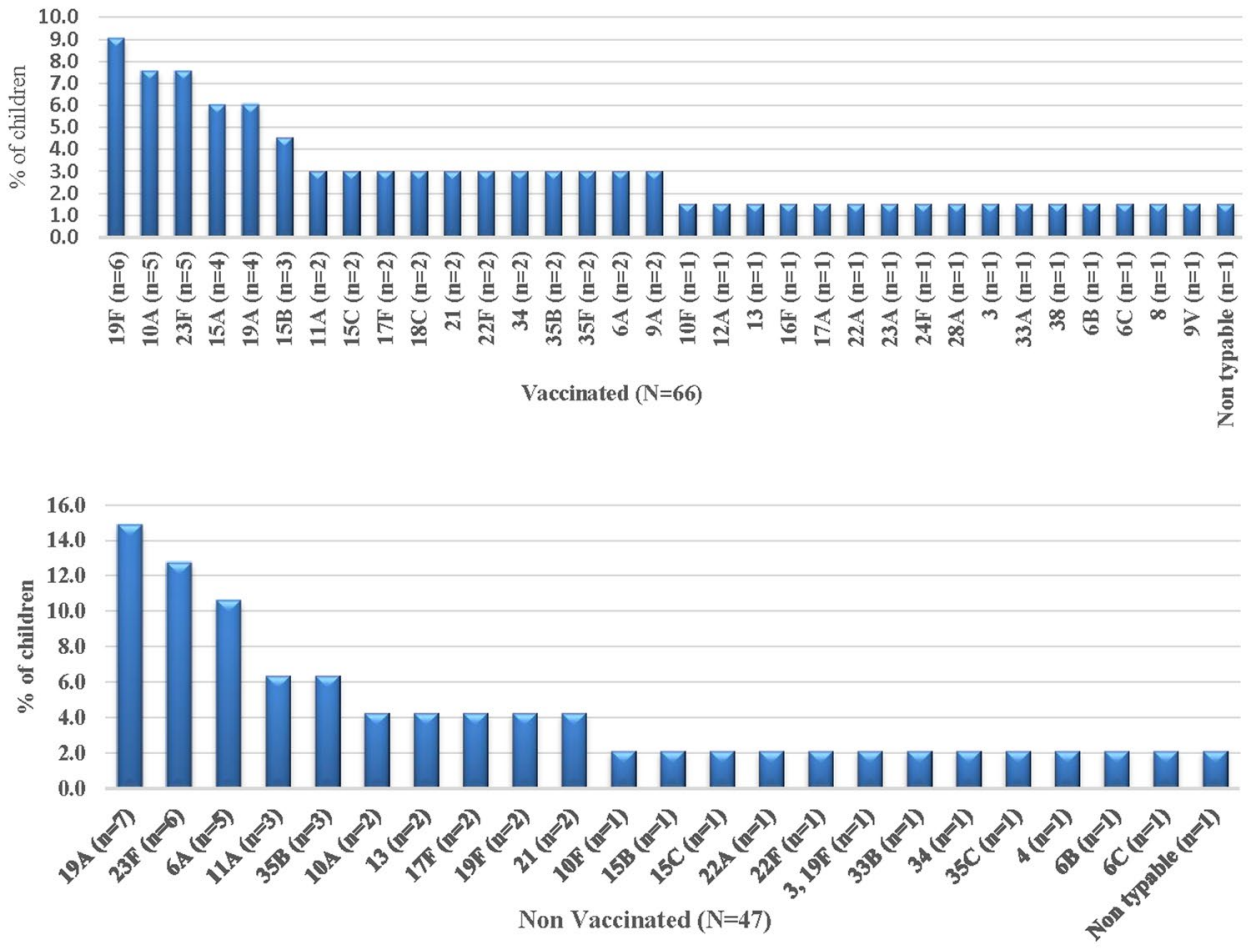
*Streptococcus pneumoniae* Serotype Distribution by PCV-13 Vaccination Status

Of 113 SP isolates belonged to 36 different serogroups/types. The vaccine serotypes were: 23F (*n* = 11), 19A (*n* = 11), 19F (*n* = 9), 6A (*n* = 7), 6B (*n* = 2), 18C (*n* = 2), 4 (*n* = 1), 3 (*n* = 1)0.11A (*n* = 5), 35B (*n* = 5), 15A (*n* = 4), 15B (*n* = 4), 17F (*n* = 4), 21 (*n* = 4), 13 (*n* = 3), 15C (*n* = 3), 22F (*n* = 3), 34 (*n* = 3), 10F (*n* = 2), 22A (*n* = 2), 35F (*n* = 2), 6C (*n* = 2), 9A (*n* = 2), 9 V (*n* = 1), 12A (*n* = 1), 16F (*n* = 1), 17A (*n* = 1), 23A (*n* = 1), 24F (*n* = 1), 28A (*n* = 1), 33A (*n* = 1), 33B (*n* = 1), 35C (*n* = 1), 38 (*n* = 1), 10A (*n* = 7), and 8 (*n* = 1). Only two isolates were non-typeable. Figures1and2 shows the distribution and frequency of serotypes by PCV vaccinated and non-vaccinated status of the children. Distribution of Vaccine and Non-vaccine serotype among all the selected hospitals are listed in Supplementary Table S1 (online data supplement).

**Fig. 3:**
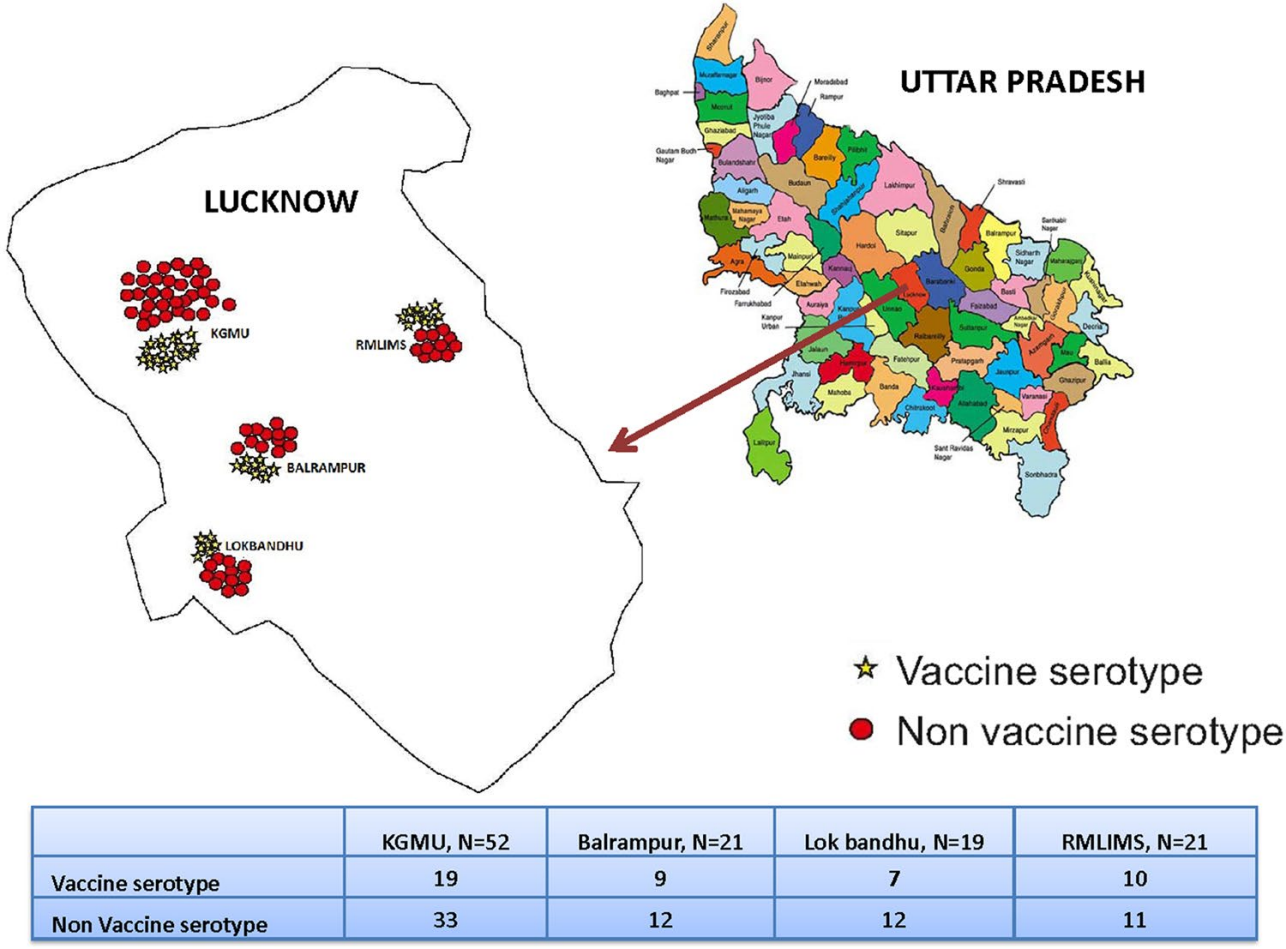
Distribution of Vaccine and Non-vaccine serotypes and spot map of the hospitals located in Lucknow

Among the PCV13 vaccinated group, 66/181 (36.46%) children were colonized with NP. Vaccine serotypes present in this group were: 18C, 19A, 19F, 23F, 3, 6A, 6B, 9 V and accounted for 33.3% (*n* = 22/66) serotypes covered in PCV13. Among the vaccine serotypes present, the three predominant serotypes were19A, 19F, 23F. Within the non-vaccinated group of children, 47/119 (39.5%) had NP colonization with SP. Vaccine serotypes in non-vaccinated group were 23F, 6A, 19A, 4, 3, 19F, and 6B, respectively, and these accounted for 48.9% (23/47) of the serotypes covered by PCV13. There was no significant difference were found in vaccine serotypes among PCV13 vaccinated and unvaccinated group (*p * = 0.09). Figure3 shows the distribution of Vaccine and Non-vaccine serotypes and spot map of the hospitals located in Lucknow.

Logistic regression model to assess the association of vaccine serotype with number of PCV doses adjusted for age is given in Table 2. There was a trend for significant reduction in vaccine serotypes in the NP with one and two or more doses (p_trend_ = 0.04). The adjusted odds ratio (AOR) of ≥ 2 doses of PCV13 with presence of vaccine serotype in the NP was 0.34 (95% CI, 0.11–1.10).

**Table 2:** Logistic regression to assess the carriage of *Streptococcus pneumoniae* serotypes with number of PCV doses adjusted for age

Variables	Vaccine serotype, *N* = 45 (%)	Non-vaccine serotype, *N* = 68 (%)	Adjusted OR (95% CI)	p-value for trend
PCV Doses				
No doses, (*N* = 47)	23 (48.9)	24 (51.1)	Reference	0.04
One doses, (*N* = 19)	9 (47.4)	10 (52.6)	0.81 (0.22–3.02)	
Two or more Doses (*N* = 47)	13 (27.7)	34 (72.3)	0.34 (0.11–1.10)	
Age in months				
24–59	9 (20)	12 (17.6)	Reference	0.5
12–23	16 (35.6)	20 (29.4)	0.32 (0.32–3.00)	
2–11	20 (44.4)	36 (52.9)	1.22 (0.34–4.44)	

A significant association of NP isolation of vaccine serotypes was found with ≥ 2 doses versus no doses of PCV13 [OR = 0.40, 95% CI (0.17–0.94) *p* = 0.036]. Adjusted for age, a tendency for this association persisted [AOR = 0.32, 95%CI (0.09–1.14) *p* = 0.08].

## Discussion

This cross-sectional study was conducted from July to August 2019 to assess the proportion of healthy children having nasopharyngeal colonization (NP) with SP. Secondary objective was to determine prevalent serotype of SP among the PCV13 vaccinated and non-vaccinated groups.

In this study, we found 37.67% colonization rate of SP in healthy children and there was no difference among those who were vaccinated with PCV13 and those that were not. The prevalence of SP carriage among healthy under-five children in India ranged from 6.5 to 69.8% [7, 24, 33–36]. Neighboring countries of India and some European countries have also reported prevalence of NP carriage of SP in healthy children between 3 and 72.9% [37–46]. Carriage rate of SP may differ depending on the ethnicity, age, environmental features, season and usage of antibiotic practices [14]. In our study, the rate of isolation of SP among children 2–11, 12–23, and 24–59 months increased with the age (35.26%, 38.04%, and 44.23%) [47].

PCV13 is effective in reducing the incidence and severity of pneumonia and other lower respiratory infections in children [3]. Therefore, as recommended by the World Health Organization, PCV 13 was introduced in 2017 in India in a phased manner as a part of routine Universal Immunization Program by the Government of India. It has been reported that high PCV13 coverage is required to interrupt VT pneumococcal transmission and achieve substantial indirect effects (to reduce the burden of vaccine type (VT) pneumococcal diseases) [48]. Near elimination of VT pneumococcal diseases has predominantly been demonstrated in countries with > 90% vaccine coverage [46]. Two observational studies from USA suggest that statistically significant indirect effects against pneumococcal VT carriage can be achieved even at 58–75% coverage among children under 5 years of age [49–51].

There was no statistically significant difference found in vaccine and non-vaccine serotype between PCV vaccinated and unvaccinated children. In a study from Netherlands, no significant changes in vaccine type IPD was reported among the vaccinated and unvaccinated children [52]. Our study has also shown that the PCV 13 vaccine schedule with the two primary doses results in significantly decreased vaccine serotype carriage in vaccinated children. It has also been reported in studies conducted in Netherlands as well as in South Africa [53–55]

PCV 13 vaccines cover approximately 40% serotypes in our study. Serotype 10A, 10F, 11A,13, 15B, 15C, 17F, 19A, 19F, 21, 22A, 22F, 23F, 3, 34, 35B, 6A, 6B, 6C, and one non-typeable were found in both vaccinated and unvaccinated children. The serotypes 19A, 23F, and 19F were most commonly reported and it represented 28% of all isolates in our study. It has been similarly observed in another study conducted by Yao et al.2011 in Mainland China [56].

### Strengths and Limitations

The study has several strengths. We compared the serotype data among PCV13 vaccinated and non-vaccinated children. Serotyping was done by Quellung method which is the gold standard method for pneumococcal capsular serotyping.

## Conclusion

More than one-third of healthy children were having a NP colonization with SP and there was no difference in vaccine serotype among vaccinated and non-vaccinated groups. Increased doses of PCV13 significantly reduces the carriage of vaccine serotype (ptrend = 0.04).

## Supplementary Information

Below is the link to the electronic supplementary material.

Supplementary file1 (DOCX 15 kb)

## Data Availability

The corresponding author has full control of all data and the data may be made available on request. All the relevant data in the manuscript.
