# Supplementary material for: Nasopharyngeal Carriage of Streptococcus pneumoniae Serotypes Among Healthy Children in Northern India
Source: Curr Microbiol. 2022 Dec 19;80(1):41. doi: 10.1007/s00284-022-03114-x (PMC9763132; doi:10.1007/s00284-022-03114-x)
Supplement: Supplementary file 1 — Supplementary file1 (DOCX 15 kb) [file 284_2022_3114_MOESM1_ESM.docx]

**Table 3: Distribution of Vaccine and Non-vaccine serotype among selected hospitals of Lucknow**

| **Site Name** | | **KGMU, N=148** | **Balrampur, N=54** | **Lok Bandhu, N=47** | **RML, N=51** | **Total** |
| --- | --- | --- | --- | --- | --- | --- |
|  | | **SP positive (n=52)** | **SP positive (n=21)** | **SP positive (n=19)** | **SP positive (n=21)** |  |
|  | **Serotypes** | **Frequency** | **Frequency** | **Frequency** | **Frequency** |  |
| **Vaccine Serotype** | 3 | 0 | 0 | 1 | 0 | 1 |
|  | 4 | 0 | 0 | 0 | 1 | 1 |
|  | 18C | 1 | 0 | 1 | 0 | 2 |
|  | 19A | 3 | 6 | 2 | 0 | 11 |
|  | 19F | 4 | 1 | 1 | 3 | 8 |
|  | 23F | 5 | 1 | 1 | 4 | 11 |
|  | 6A | 5 | 1 | 1 | 0 | 7 |
|  | 6B | 1 | 0 | 0 | 1 | 2 |
|  | 9V | 0 | 0 | 0 | 1 | 1 |
| **Non-vaccine Serotype** | 8 | 1 | 0 | 0 | 0 | 1 |
|  | 13 | 2 | 0 | 0 | 1 | 3 |
|  | 21 | 2 | 1 | 0 | 1 | 3 |
|  | 34 | 2 | 0 | 1 | 0 | 3 |
|  | 38 | 1 | 0 | 0 | 0 | 1 |
|  | 10A | 1 | 3 | 2 | 1 | 7 |
|  | 10F | 1 | 0 | 1 | 0 | 2 |
|  | 11A | 4 | 0 | 1 | 0 | 5 |
|  | 12A | 0 | 0 | 1 | 0 | 1 |
|  | 15A | 2 | 0 | 2 | 0 | 4 |
|  | 15B | 1 | 3 | 0 | 0 | 4 |
|  | 15C | 1 | 2 | 0 | 0 | 3 |
|  | 16F | 1 | 0 | 0 | 0 | 1 |
|  | 17A | 1 | 0 | 0 | 0 | 1 |
|  | 17F | 2 | 1 | 0 | 1 | 4 |
|  | 22F | 1 | 1 | 1 | 2 | 5 |
|  | 23A | 0 | 0 | 1 | 0 | 1 |
|  | 24F | 1 | 0 | 0 | 0 | 1 |
|  | 28A | 1 | 0 | 0 | 0 | 1 |
|  | 3,19F | 1 | 0 | 0 | 0 | 1 |
|  | 33A | 0 | 0 | 0 | 1 | 1 |
|  | 33B | 0 | 0 | 1 | 0 | 1 |
|  | 35B | 2 | 1 | 1 | 1 | 5 |
|  | 35C | 0 | 0 | 0 | 1 | 1 |
|  | 35F | 2 | 0 | 0 | 0 | 2 |
|  | 6C | 1 | 0 | 0 | 1 | 2 |
|  | 9A | 1 | 0 | 0 | 0 | 1 |
|  | Non-Typable | 1 | 0 | 0 | 1 | 2 |
